# Validity and reliability study of the Turkish version of the Empathy Quotient- 8 in Turkish university students

**DOI:** 10.1186/s41155-025-00344-3

**Published:** 2025-04-08

**Authors:** Mehmet Emin Turan, Erkan Turan, Mustafa Açar, Izaddin Ahmad Aziz, Abdulmohsen Mohammed Abdullah Alkhulayfi, Hicham Khabbache, Amelia Rizzo, Francesco Chirico, Juan Gómez-Salgado, Murat Yıldırım

**Affiliations:** 1 Department of Psychology, Ağrı Ibrahim Cecen University, Ağrı, Türkiye; 2 Turkish Ministry of National Education, Istanbul, Türkiye; 3 Turkish Ministry of National Education, Gaziantep, Türkiye; 4 Psychological and Educational Counselling Department, College of Education, Salahaddin University-Erbil, Erbil, Iraq; 5 English Department, College of Education, Bayan University, Erbil, Iraq; 6 Department of Business Administration, Faculty of Economics and Administration, King Abdulaziz University, Jeddah, Saudi Arabia; 7 Department of Psychology, Faculty of Arts, Human Sciences Fès-Saïss, Sidi Mohamed Ben Abdellah University, Fez, Morocco; 8 Department of Clinical and Experimental Medicine, University of Messina, Messina, Italy; 9 Department of Cognitive Sciences, Psychological, Educational, and Cultural Studies, University of Messina, Messina, Italy; 10 Post-Graduate School of Occupational Health, Università Cattolica del Sacro Cuore, Rome, Italy; 11 Health Service Department, Ministry of the Interior, Italian State Police, Milan, Italy; 12 Department of Sociology, Social Work and Public Health, Faculty of Labour Sciences, University of Huelva, Huelva, Spain; 13 Safety and Health Postgraduate Program, Universidad Espíritu Santo, Guayaquil, Ecuador; 14 Department of Psychology, Faculty of Science and Letters, Agri Ibrahim Cecen University, Fırat Mahallesi Yeni Üniversite Caddesi No: 2 AE/1 04100 Merkez, Ağrı, Türkiye; 15 Psychology Research Center, Khazar University, Baku, Azerbaijan

**Keywords:** Empathy, Validity, Reliability, Turkish university students

## Abstract

**Background:**

Empathy is an important psychological construct that plays a significant role in social interactions, mental health, and well-being. Despite the significance of empathy in psychological research and mental health, there is a lack of validated and concise measures available in Turkish.

**Objective:**

This study aims to evaluate the psychometric properties of the eight-item Empathy Quotient (EQ- 8) in Turkish university students, assessing its reliability, convergent validity, and factor structure. Therefore, we seek to determine its suitability for use in psychological and mental health research within Turkish-speaking populations.

**Methods:**

We collected the data from two groups. The data of the first group (N = 198) was used to test the factor structure of the EQ- 8 by randomly splitting the data into two halves. The first half was used for the exploratory factor analysis (EFA), and the second half was used for confirmatory factor analysis (CFA), while the second group (N = 47) was carried out to test the reliability of EQ- 8.

**Results:**

The results of the EFA and CFA yielded a one-factor solution for the EQ- 8. The internal consistency reliability was found to be good in both subsamples. Test–retest reliability was established as.86. As to the convergent validity, the scores on EQ- 8 were significantly positively related to the scores of the Toronto Empathy Questionnaire.

**Conclusions:**

These results provide support for the Turkish version of the EQ- 8 as a psychometrically sound instrument for measuring empathy. These results contribute to cross-cultural research and the evaluation of interventions targeting empathy.

## Introduction

Nowadays, with globalization's influence, individuals can interact more with each other virtually and in real-time. Interactions between individuals from different cultural, social, and racial backgrounds can cause misunderstandings. These misunderstandings can negatively affect individuals'psychological health (Gulin, 2020; Manczak et al., 2016). Empathy is one of the critical psychological constructs for understanding differences and promoting the psychological health of individuals (Erskine et al., 2022).

Lately, there has been a growing interest in measuring and understanding empathy, which is an essential construct for promoting understanding, compassion, and positive change in a fast-paced, interconnected world where many challenges and opportunities exist (Erskine et al., 2022; Nembhard et al., 2022). Therefore, empathy can be considered a critical psychological ingredient to positive psychological health. Appropriate application of empathy contributes to developing a more inclusive, harmonious, and collaborative society, better positioned to address the intricate challenges of our era (Hong & Han, 2020).

Empathy is understanding and sharing another person's feelings, thoughts, and experiences. It involves connecting emotionally with others and seeing the world from their perspective. Empathy is an essential aspect of human social interaction (Bas-Sarmiento, 2020; Nembhard et al., 2022). It plays a vital role in establishing and maintaining relationships, allowing individuals to relate to and support each other emotionally. Empathy is valuable in various aspects of life, including personal relationships, parenting, healthcare, and professional environments (Bas-Sarmiento, 2020; Hong & Han, 2020). It will promote better communication, conflict resolution, and understanding of the people around. Like other psychological constructs such as similar to other positive psychological constructs such as resilience (Ashraf et al., 2023; Turan, 2021a), happiness (Yıldırım & Maltby, 2022), expressivity (Turan, 2021b), social support (Yildirim et al., 2020), coping (Kızılgeçit & Yıldırım, 2023), psychological capital (Çağış & Yıldırım, 2023), personal growth initiative (Green & Yıldırım, 2022), hope (Yıldırım et al., 2022), and self-esteem (Yildirim et al., 2019), cultivating and practising empathy can lead to more meaningful and satisfying life, better mental health and well-being outcomes as well as connections with others, contributing to a more compassionate and understanding society (Johnston, 2023).

Measuring empathy is difficult due to its multifaceted nature of empathy. Empathy is a complex psychological construct that some techniques have been developed to measure empathy like self-report (Baron-Cohen & Wheelwright, 2004), behavioural (Cliffordson, 2001), physiological (Sassenrath et al., 2020), and neuroimaging (Tholen et al., 2020) techniques. One of the frequently used self-report empathy models is the Empathy Quotient (EQ) measurement model.

Empathy is a complex concept with both cognitive and affective components. The conceptualization of the EQ acknowledges the multifaceted nature of empathy, including both cognitive and affective elements. However, the EQ assigns a total empathy score, recognizing that cognitive and affective components often co-occur and are challenging to separate in most situations (Baron-Cohen & Wheelwright, 2004).

The EQ is a self-report instrument designed to measure empathy in individuals, particularly those with autism spectrum disorders (Baron-Cohen & Wheelwright, 2004). The psychometric properties of the EQ have also been demonstrated in other studies. For example, Lawrence et al. (2004) found that the EQ has evidence of validity and reliability. Lawrence et al. (2004) reported that the EQ has evidence of high test–retest reliability, factorial validity (3 factors labelled ‘cognitive empathy’, ‘emotional reactivity’, and ‘social skills’ respectively), association with a non-verbal mental state inference test (Eyes Task), and Interpersonal Reactivity Index subscales (empathic concern and perspective-taking), suggesting concurrent validity. The EQ is frequently used in psychological research and clinical settings to assess an individual's ability to understand and respond to the emotions of others. The EQ was initially a 60-item scale developed by Baron-Cohen and Wheelwright (2004). Subsequently, Wakabayashi et al. (2006) shortened the EQ to 22 items, and Loewen et al. (2010) further refined it to 8 items. Loewen et al. (2010) demonstrated that both the 22-item and 8-item versions exhibit satisfactory psychometric properties, establishing the EQ- 8 as a valid and reliable instrument. The EQ, along with its abbreviated formats, has found application in diverse studies (Lima & Osório, 2021).

Validity and reliability studies of the EQ- 8 have also been conducted in other languages, such as in Greek by Kokkinos et al. (2014) and Spanish by Frías-Armenta et al. (2021), demonstrating good psychometric properties of the EQ- 8. The application of the EQ- 8 has been tested across diverse studies, ranging from topics like COVID- 19 (Stoler et al., 2022; Frías-Armenta et al., 2021), romantic infidelity (Wilkinson & Dunlop, 2020), human capabilities (Chan et al., 2019), to the assessment of counsellor empathy (Johnson et al., 2020).

As mentioned above, empathy is a critical concept studied in many scientific disciplines. In this regard, a functional measurement tool that evaluates empathy may facilitate the way for comparison of better research outcomes. Despite the evidence regarding the psychometric properties of the EQ- 8 in many cultures (Chan et al., 2019; Kokkinos et al., 2014; Stoler et al., 2022), to the best of our knowledge, the EQ- 8 in Turkish has not yet been validated in Turkish culture. Therefore, this study aims to fill this gap by focusing on the Turkish adaptation of the EQ- 8. Based on this aim, we created the following hypotheses.

- H1: The EQ- 8 would demonstrate good internal consistency and test–retest reliabilities.
- H2: The EQ- 8 would have a one-factor structure.
- H3: The EQ- 8 would be significantly positively related to other existing empathy questionnaires.

## Method

### Participants and procedure

We used the convenience sampling method to collect data. We collected data from two groups. The data of the first group was collected from 198 participants (98 female). Among the 198 participants, 137 were aged 18–22, 43 fell within the 23–28 age range, and 18 were 29 years old or above. The data of the second group (n = 47) was also collected from students and used to assess the test–retest reliability of the EQ- 8. All participants were university students. Ethics committee approval was obtained from the first author’s University Ethics Committee (reference number: E- 95531838–050.99–18,530). In addition, every stage of the study was carried out in accordance with the Declaration of Helsinki. Data was collected voluntarily through an online data collection software using Google Forms. Data collection was conducted between February 2022 and May 2022. Information about the study group is presented in Table 1.

**Table 1:** Demographic information

		EFA Group		CFA Group		Test–Retest Group	
Variable	Level	N	%	N	%	N	%
Gender	Female	45	42,9	53	50,5	35	74,5
	Male	60	57,1	52	49,5	12	25,5
Age	18–22 years old	66	62,9	71	67,6	24	51,1
	23–28 years old	25	23,8	18	28,6	23	48,9
	29 years and above	14	13,3	4	3,8	-	-
Perceived financial status	Low	28	26,7	20	19	13	27,7
	Middle	74	69,5	82	78,1	30	63,8
	High	3	3,8	3	2,9	4	8,5
Grade point average	1.99 and below	4	3,8	3	2,9	2	3,43
	Between 2.00–2.50	8	7,6	8	7,6	3	6,4
	Between 2.51–3.00	34	32,4	50	47,6	18	38,3
	Between 3.01–3.50	43	41,0	37	35,2	16	34,0
	Between 3.51–4.00	16	15,2	7	6,7	8	17,0

### Measures

#### Empathy Quotient—8 (EQ- 8; Loewen et al., 2010)

The EQ- 8 assesses empathy through statements such as “I find it easy to put myself in somebody else's shoes”, which respondents rate on a 4-point scale. For each item, a scoring system assigns 2 points for responses indicating"strongly agree,"1 point for those expressing"slightly agree,"and 0 points for both"slightly disagree"and"strongly disagree"on items 1 through 4. Conversely, allocate 2 points for answers reflecting"strongly disagree,"1 point for"slightly disagree,"and 0 points for both"slightly agree"and"strongly agree"on items 5 through 8. Higher scores indicate greater empathy. The scale demonstrated good reliability, with a Cronbach’s alpha coefficient of 0.89 in this study.

#### Toronto Empathy Questionnaire (TEQ; Spreng et al., 2009)

The TEQ is a self-report scale developed to assess empathy as a primarily emotional process. The TEQ Turkish version includes 13 items (e.g.,"Other people’s misfortunes do not disturb me a great deal “). Each item was rated on a 5-point Likert scale ranging from 0 (never) to 4 (always). A higher score on the scale represents a higher level of empathy. The Turkish version of the TEQ showed good evidence of reliability and validity (Totan et al., 2012). The Cronbach’s alpha coefficient for the TEQ in this study was 0.88 in the total sample.

### Translation

The Turkish adaptation study started with obtaining the permission of Peter John Loewen, who developed the EQ- 8 form to adapt the scale. The measurement tool for assessing empathy was translated into Turkish from its original English version. Following the translation process, the items were back-translated to ensure that the meaning between the original and translated texts remained equivalent. The translation of the EQ- 8 was carried out by three experts who have a good knowledge of English and Turkish. A trial form of the translation has been prepared. Researchers evaluated the instrument translated into Turkish to assess and improve reliability and validity before the beginning of the study. This form was reviewed and approved by three faculty members who are experts in the field of counselling and psychology.

### Statistical analysis

The construct validity of the EQ- 8 was examined with Exploratory Factor Analysis (EFA) and Confirmatory Factor Analysis (CFA). The structure obtained as a result of EFA was tested with CFA. CFA model fit was examined with CMIN/df, CFI, GFI, and RMSEA fit indices. The convergent validity of the EQ- 8 was examined with the TEQ. The reliability of the EQ- 8 was examined using the test–retest method, McDonald’s ω, and Cronbach’s α internal consistency reliability coefficient. McDonald’s ω and Cronbach’s α internal consistency reliability coefficients of the EQ- 8 were calculated in both EFA and CFA samples. Item analysis and corrected item-total correlations of the EQ- 8 were also examined. Statistical analyses were performed with AMOS (used to compute the CFA), jamovi 2.2.5 (used to compute the McDonald’s ω coefficient), and SPSS 26.0 (used for other whole analyses) programs. Widely accepted fit indices regarding model fit were considered as a good fit of ≤ 3 for CMIN/DF, ≥ 0.90 for CFI, ≤ 0.08 for RMSEA, and ≥ 0.95 for GFI (Hu & Bentler, 1999; Kline, 2005; Tabachnick & Fidell, 2001).

## Results

### Preliminary analysis

For this study, data were collected from two groups (Group 1 = 198; Group 2 = 47). The first group's data were used for Exploratory Factor Analysis (EFA) and Confirmatory Factor Analysis (CFA), while test–retest reliability was assessed using data from 47 participants in Group 2. Group 1 was randomly divided into two equal subsamples: EFA was conducted with 105 participants (45 female), and CFA was performed with the remaining 105 participants (53 female). A widely accepted guideline for determining sample size suggests a cases-per-variable ratio between 4:1 and 20:1 (Bentler & Chou, 1987; Tanaka, 1987). Following this criterion, our sample size for both EFA and CFA meets the recommended threshold, with a participant-to-item ratio of 105:8, exceeding the commonly suggested minimum of 10:1.

### Exploratory factor analysis

An Exploratory Factor Analysis (EFA) of the EQ- 8 was conducted on 105 participants. First of all, to evaluate the data's suitability for factor analysis, the Kaiser–Meyer–Olkin (KMO) coefficient and Bartlett's Sphericity Test were performed. The analysis showed that the KMO sample suitability coefficient was 0.85, and the χ2 value in Bartlett's Sphericity Test was 410.076 (*p* < 0.001). These findings show that the data to be used for EFA are appropriate. Then, EFA was conducted on eight items using a Principal Components Analysis without any rotation. As a result of the analysis, a structure with eight factors was obtained. However, for the determined factors to be meaningful, their eigenvalues must be above 1.00. Additionally, these factors should be graphed in the line chart. As a result of EFA, the eigenvalues of seven of the eight factors of the EQ- 8 are below"1.00". Therefore, factors with eigenvalues below"1.00"were not considered sub-dimensions. The eigenvalues of the EQ- 8 with eigenvalues below"1.00"range between 0.964 and 0.225. In this context, the items of the EQ- 8 are collected under a single factor. This single-factor structure, with an eigenvalue of 4.468, explains 55.85% of the total variance.

**Fig. 1:**
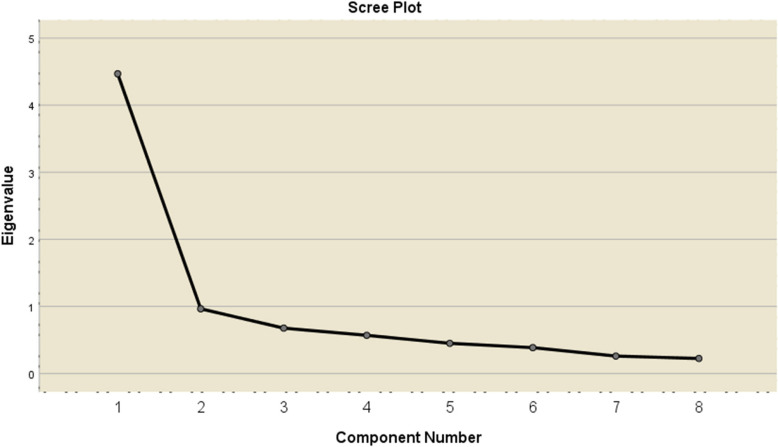
Scree plot graph for the EQ- 8 Scale

Furthermore, the Scree plot in Fig. 1 was analysed, revealing a distinct break after the first factor. Therefore, the EQ- 8 was treated as a single-factor scale. The factor loading values from the EFA ranged between 0.66 and 0.81. Fig. 1 illustrates the Scree Plot for the EQ- 8, while Table 2 provides an overview of the EQ- 8 results.

**Table 2:** The results of EFA

Item	Factor loadings	Explained variance	Eigen value
Item 1	.66	55.85	4.47
Item 2	.80		
Item 3	.74		
Item 4	.81		
Item 5	.78		
Item 6	.75		
Item 7	.70		
Item 8	.73		

### Confirmatory factor analysis

**Fig. 2:**
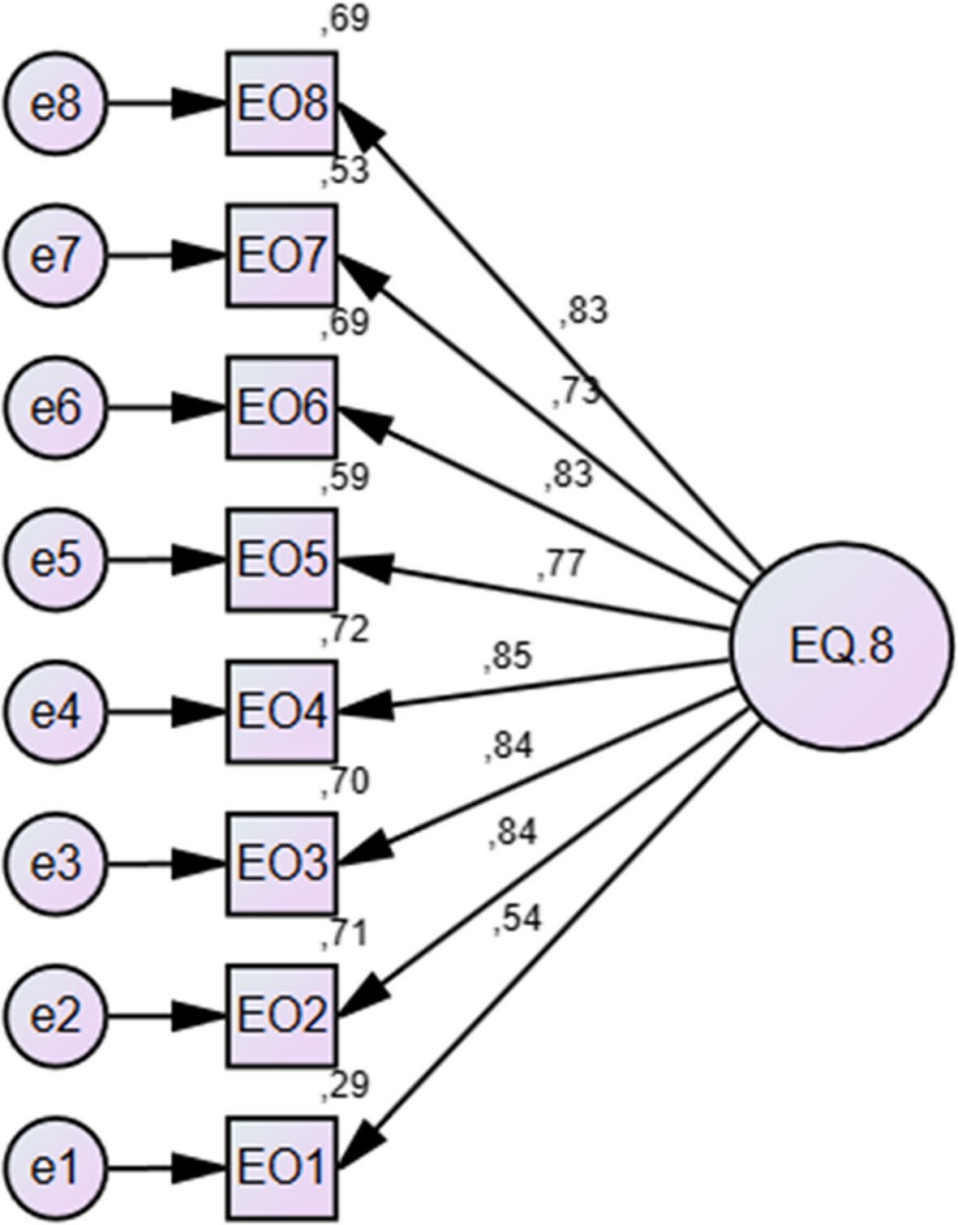
The results of the CFA model being tested for the EQ- 8 Scale

The single-factor structure of the EQ- 8 was confirmed through confirmatory factor analysis (CFA) conducted on data from 105 participants. The analysis revealed that the 8-item, one-dimensional EQ- 8 model exhibited good fit indices. (CMIN = 31.804, df = 20, CMIN/DF = 1.590, *p* < 0.05, RMSEA = 0.07, CFI = 0.93, GFI = 0.98). The standardized factor loadings are shown in Fig. 2.

### Convergent validity

The convergent validity of the EQ- 8 was examined with the TEQ. The EQ- 8 and the TEQ were applied to the EFA and CFA groups. Pearson product-moment correlation Analysis examined the relationship between the TEQ and the EQ- 8. As a result, the analysis showed that the EQ- 8 was highly positively correlated with the TEQ (EFA sample: *r* = 0.78**; CFA sample: *r* = 0.77**, **p < 0.001).

### Reliability

The reliability of the EQ- 8 was examined using internal consistency (Cronbach α, McDonald ω) and test–retest methods. Cronbach’s α and McDonald’s ω internal consistency reliability coefficients of the EQ- 8 were calculated in two different samples, the EFA sample and the CFA sample. The Cronbach’s α internal consistency reliability coefficient of the EQ- 8 was calculated as 0.88 in the EFA sample and 0.90 in the CFA sample. The McDonald’s ω internal consistency reliability coefficient of the EQ- 8 was calculated as 0.89 in the EFA sample and 0.91 in the CFA sample. Whether the EQ- 8 gave consistent results at different times was examined using the test–retest method. Test–retest analysis was carried out on two applications collected three weeks apart from 47 individuals. The correlation coefficient between the two applications was found to be 0.86.

### Item analysis

The discriminatory power of the EQ- 8 items was examined by item analysis. Item analysis was examined separately on both EFA and CFA samples. In this context, the corrected item-total correlation coefficients of the EQ- 8 items were examined. The analysis showed that the corrected item-total correlation coefficients of the scale varied between 0.56 and 0.73 in the EFA sample and between 0.52 and 0.77 in the CFA sample. Findings regarding the EQ- 8 item analysis are presented in Table 3.

**Table 3:** Findings regarding the item analysis of the EQ- 8 Scale

EFA Sample		CFA Sample	
Item number	r_jx_	Item number	r_jx_
Item 1	.58	Item 1	.52
Item 2	.72	Item 2	.77
Item 3	.64	Item 3	.70
Item 4	.73	Item 4	.72
Item 5	.70	Item 5	.66
Item 6	.67	Item 6	.76
Item 7	.59	Item 7	.69
Item 8	.63	Item 8	.73

r_jx_ = item discrimination index

## Discussion

Lack of empathy threatens individuals'interpersonal relationships and is a significant risk factor associated with psychological well-being worldwide. Much research has focused on understanding the relationship between empathy and psychological health (Choi et al., 2016; Manczak et al., 2016; Nembhard et al., 2022). Researchers and experts need an up-to-date and short measurement tool to evaluate empathy. This study was carried out to meet this need. This study aims to test the Turkish psychometric properties of the EQ- 8. As mentioned above, this study aimed to adapt the EQ- 8 Form to Turkish and conduct reliability and validity analysis studies of the scale. Thus, the need for a short measurement tool tested in Turkish culture to evaluate empathy will be met.

The scale's exploratory factor analysis, confirmatory factor analysis, and convergent validity analyses were examined in this regard. The scale's reliability was evaluated with internal consistency, test–retest, and item-total correlations. Analyses performed to evaluate reliability indicate that the scale can be considered reliable. The reliability coefficient of measurement tools used in scientific research is recommended to be 0.70 and above. The test–retest reliability coefficient should be 0.80 and above. For item-total correlations, it is stated that scale items'loadings of 0.30 and above are distinctive (Büyüköztürk, 2012; Tabachnick & Fidell, 2001). The findings are discussed below.

The present study examined the psychometric properties of the Turkish version of the EQ- 8. The results show that the EQ- 8 is a psychometrically sound scale measuring empathy in Turkish society. The results of exploratory and confirmatory factor analyses revealed a single-factor solution consisting of 8 items with high internal consistency reliability. Analyses conducted to evaluate validity indicate that the scale can be considered valid. Exploratory factor analysis revealed a structure of 8 items and a single dimension, explaining 55.85% of the total variance. Confirmatory factor analysis results show that the model produces good fit values. These findings are consistent with the study in which the EQ- 8 was originally developed. Loewen et al. (2010) reported that the EQ- 8 consists of the eight items with the highest factor loadings from the original EQ (60-item) and has evidence of reliable internal validity (α = 0.76). Wilkinson and Dunlop (2020) also found high internal consistency reliability evidence (α = 0.82).

Additionally, the relationship between the EQ- 8 and the TEQ was examined within the scope of convergent validity analysis. Convergent validity analysis results showed that the EQ- 8 scores were statistically significantly related to the TEQ (*p* < 0.001). These results provide evidence of the scale's convergent validity. Spreng et al.. (2009) found that the TEQ correlated positively with the EQ (r = 0.80, *p* < 0.001), in parallel with the findings of the present study.

### Contribution of the study

The study contributes to the existing literature by establishing the Turkish version of the EQ- 8 as a reliable and valid measurement tool. The demonstrated adequate reliability values and evidence from analyses support the value of the EQ- 8 for use in scientific research. Consequently, the Turkish version of the EQ- 8 emerges as a validated measurement tool, offering both researchers and practitioners a reliable instrument in areas such as positive psychology, counselling, and social work.

#### Limitations

The current study has some limitations. First, the results of this study were based on self-reported data. In future studies, different methods, such as implicit measurements, can be used to prevent individuals from reacting in the direction of social desirability. Secondly, a key limitation of this study is that the sample consists exclusively of university students, which may limit the generalizability of the findings to broader populations. As a result, the sample is not fully representative of the general population. Future research should aim to replicate these findings using more diverse samples, including individuals from different age groups and educational backgrounds to enhance the external validity of the scale. Additionally, this research was conducted on the Turkish population. This study can be repeated with the participation of individuals living in countries other than Turkey, living in different socio-cultural environments, and clinic and non-clinic samples. In this regard, researchers can evaluate the results of the adapted measurement tool in different cultural contexts. The results obtained may also contribute to detecting cultural similarities and different patterns between regions. The final limitation of this study is that criterion validity was assessed using only the Toronto Empathy Questionnaire as the sole variable. While this provides useful evidence, testing criterion validity with additional concepts could present a better evaluation. Future research should consider including multiple variables to strengthen the understanding of the validity of the scale.

## Conclusion

Despite all these limitations, the findings showed that the Turkish version of the EQ- 8 has very good reliability and validity. The validation of the EQ- 8 shows that this scale can be used in many theoretical and empirical studies on empathy in the relevant literature. Additionally, this scale will allow educators, counsellors, psychologists, researchers, and policymakers to adapt and evaluate the effectiveness of empathy cultivation interventions. In conclusion, the Turkish validation of the EQ- 8 showed good psychometric properties, indicating adequate internal consistency reliability and good single-factor structure. Therefore, in future studies, the Turkish version of the EQ- 8 is a reliable measurement tool for assessing empathy for young adults.

## Data Availability

The datasets generated during and/or analysed during the current study are available from the corresponding author upon reasonable request.

## Abbreviations

EQ- 8: Empathy Quotient- 8
EFA: Exploratory factor analysis
CFA: Confirmatory factor analysis
TEQ: Toronto Empathy Questionnaire
CMIN/DF: Chi-square divided by Degrees of Freedom.
RMSEA: Root Mean Square Error of Approximation.
CFI: Comparative Fit Index.
GFI: Goodness of Fit Index
